# Supplementary material for: From sequence to enzyme mechanism using multi-label machine learning
Source: BMC Bioinformatics. 2014 May 19;15:150. doi: 10.1186/1471-2105-15-150 (PMC4229970; doi:10.1186/1471-2105-15-150)
Supplement: Additional file 2 — Java code of ml2db. Additional file ml2db_code.tar.gz contains the Java source code to run the multi-label machine learning experiments and save the results to database. The code’s Javadoc is included. [file 1471-2105-15-150-S2.zip › additional file 2/ml2db/utils/doc/uk/ac/ed/inf/utils/database/TableRowSaver.html]

TableRowSaver


---


|  |  |  |
| --- | --- | --- |
| **Overview** \| **Package** \| **Class** \| **Use** \| **Tree** \| **Deprecated** \| **Index** \| **Help** | |  |
| **PREV CLASS**   **NEXT CLASS** | **FRAMES**    **NO FRAMES**     **All Classes** |  |
| SUMMARY: NESTED \| FIELD \| CONSTR \| METHOD | DETAIL: FIELD \| CONSTR \| METHOD |  |


---


## uk.ac.ed.inf.utils.database Class TableRowSaver

```
java.lang.Object
  uk.ac.ed.inf.utils.database.TableRowSaver
```

---

``` public class TableRowSaver extends java.lang.Object ```

Outputs a row of data to screen, file and database.

**Version:**
:   15 Aug 2010

**Author:**
:   Luna De Ferrari luna.deferrari-at-ed.ac.uk

---

**Constructor Summary**

`TableRowSaver(Table table)`

`TableRowSaver(TableRow tableRow)`


| **Method Summary** | |
| --- | --- |
| `java.lang.String` | `dataToString(TableRow row, java.lang.String filePath)` |
| `TableRow` | `fromValuesToRow(java.lang.Object[] values)`             Only works if the array contains all the values needed for the row and compatible with the row columns in the table object! TODO implement checks |
| `TableRow` | `getCurrentRow()`             Get the last table row saved or being saved |
| `Table` | `getTable()` |
| `TableManager` | `getTableManager()` |
| `int` | `saveAndPrintRow(java.lang.Object[] values, java.lang.String filePath)` |
| `int` | `saveAndPrintRow(TableRow row, java.lang.String filePath)` |
| `int` | `saveRowToTable(TableRow row)` |

**Methods inherited from class java.lang.Object**

`equals, getClass, hashCode, notify, notifyAll, toString, wait, wait, wait`

**Constructor Detail**

### TableRowSaver

```
public TableRowSaver(Table table)
```

---


### TableRowSaver

```
public TableRowSaver(TableRow tableRow)
```


**Method Detail**

### dataToString

```
public java.lang.String dataToString(TableRow row,
                                     java.lang.String filePath)
```

---


### fromValuesToRow

```
public TableRow fromValuesToRow(java.lang.Object[] values)
```

:   Only works if the array contains all the values needed for the row and
    compatible with the row columns in the table object! TODO implement
    checks

    :   **Parameters:**: `values` - **Returns:**

---


### getCurrentRow

```
public TableRow getCurrentRow()
```

:   Get the last table row saved or being saved

---


### getTable

```
public Table getTable()
```

---


### getTableManager

```
public TableManager getTableManager()
```

---


### saveAndPrintRow

```
public int saveAndPrintRow(java.lang.Object[] values,
                           java.lang.String filePath)
```

---


### saveAndPrintRow

```
public int saveAndPrintRow(TableRow row,
                           java.lang.String filePath)
```

---


### saveRowToTable

```
public int saveRowToTable(TableRow row)
```


---


|  |  |  |
| --- | --- | --- |
| **Overview** \| **Package** \| **Class** \| **Use** \| **Tree** \| **Deprecated** \| **Index** \| **Help** | |  |
| **PREV CLASS**   **NEXT CLASS** | **FRAMES**    **NO FRAMES**     **All Classes** |  |
| SUMMARY: NESTED \| FIELD \| CONSTR \| METHOD | DETAIL: FIELD \| CONSTR \| METHOD |  |


---
